# Parallel Routes of Human Carcinoma Development: Implications of the Age-Specific Incidence Data

**DOI:** 10.1371/journal.pone.0007053

**Published:** 2009-09-23

**Authors:** James P. Brody

**Affiliations:** Department of Biomedical Engineering University of California Irvine, Irvine, California, United States of America; University of East Piedmont, Italy

## Abstract

**Background:**

The multi-stage hypothesis suggests that cancers develop through a single defined series of genetic alterations. This hypothesis was first suggested over 50 years ago based upon age-specific incidence data. However, recent molecular studies of tumors indicate that multiple routes exist to the formation of cancer, not a single route. This parallel route hypothesis has not been tested with age-specific incidence data.

**Methodology/Principal Findings:**

To test the parallel route hypothesis, I formulated it in terms of a mathematical equation and then tested whether this equation was consistent with age-specific incidence data compiled by the Surveillance Epidemiology and End Results (SEER) cancer registries since 1973. I used the chi-squared goodness of fit test to measure consistency. The age-specific incidence data from most human carcinomas, including those of the colon, lung, prostate, and breast were consistent with the parallel route hypothesis. However, this hypothesis is only consistent if an immune sub-population exists, one that will never develop carcinoma. Furthermore, breast carcinoma has two distinct forms of the disease, and one of these occurs at significantly different rates in different racial groups.

**Conclusions/Significance:**

I conclude that the parallel route hypothesis is consistent with the age-specific incidence data only if carcinoma occurs in a distinct sub population, while the multi-stage hypothesis is inconsistent with this data.

## Introduction

The multi-stage hypothesis [1], [2] states that cancers develop through a series of genetic alterations. This hypothesis is schematically indicated by the following diagram,
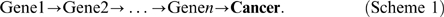
Alterations occur successively in 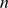 genes (Gene1, Gene2, 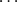, Gene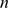) before a tissue specific cancer develops. The process is best studied in human colorectal cancers, where the first four genes in the sequence have been identified as *APC*, *K-ras*, *DCC*, and *p53* [2].

The multi-stage hypothesis was first suggested over 50 years ago based upon an analysis of the age-specific incidence data [3], [4]. This data consists of a histogram of the age at which a population develops cancer. It has long been interpreted to suggest that four to six rate-limiting events are required for the formation of cancer.

Some problems exist with the multi-stage hypothesis. The sequence *APC*, *K-ras*, *DCC*, and *p53* is not the only route to developing colon cancer. This particular route accounts for a subset: only about half of colorectal cancer patients have detectable mutations in the *APC* gene [5], and alternative routes have been identified that do not involve *APC* [6].

Anomalies also exist with the age-specific incidence data that cannot be explained by the multi-stage hypothesis. The incidence for several carcinomas drops at advanced ages [7], [8]. Breast carcinoma incidence data is very different than the other carcinomas [9], and it varies depending on the race and nationality of the population studied [10]. Prostate carcinoma incidence increases much more rapidly with age than other carcinomas, implying that 20 to 30 mutations are required for its development.

Based on several molecular studies [11], [12], it is now generally accepted that multiple parallel routes exist to the formation of tumors, as indicated by the following diagram:
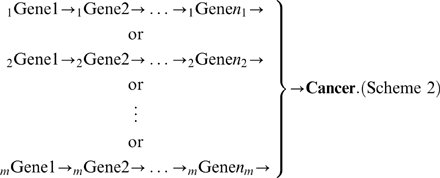



This **parallel route hypothesis** is a generalization of the multi-stage hypothesis. The number of routes, 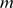, and the number of genes involved in each route, 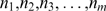 are not known for any cancer. Just as the multi-stage hypothesis was tested against the age-specific incidence data, so too can the parallel route hypothesis.

Mathematical models of the age-specific incidence of cancer have been an important tool to understand the tumorigenesis process [13], [14]. For instance, Knudson's two-hit hypothesis of retinoblastoma age-incidence data led to the identification of the first tumor suppressor gene, *Rb1* [15]. One of the few pieces of evidence about the human carcinogenesis process is epidemiological data on the incidence as a function of age [16].

I tested the parallel routes hypothesis by comparing a mathematical representation of it to the age-specific incidence data for different forms of cancer. Following this, I examined the implications of the hypothesis and attempted to better understand some of the anomalies of the age-specific incidence data.

## Results and Discussion

I tested the validity of the parallel routes hypothesis with the most powerful dataset available, the Surveillance Epidemiology and End Results 17 registries (SEER-17) data collected in the year 2000 for the age-specific incidence of colon carcinoma. The year 2000 data monitored 73 million people in the United States and recorded over 22,000 cases of colon carcinoma. The population under surveillance, an important factor, was directly measured by the 2000 US Census data. Thus, this is the best data to use to test this hypothesis.

**Figure 1:**
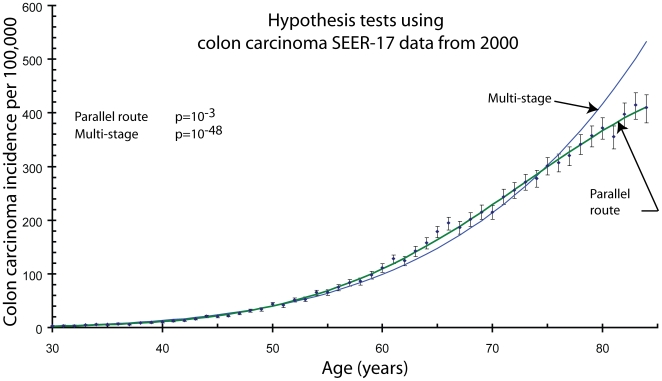
This graph compares two different hypotheses for the development of carcinoma with the age-specific incidence data. The multi-stage hypothesis, as represented by the Armitage-Doll model, Equation 2 [51], and parallel route hypothesis, represented by Equation 3. The Armitage-Doll model is an approximation of the differential form of the Poisson process, Equation 1. Using this equation gives 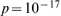, a substantially better, but still clearly unacceptable fit. Thus, the parallel route hypothesis is clearly acceptable, while the multi-stage hypothesis is not. The error bars represent 95% confidence intervals in the measured values.

The parallel route hypothesis is consistent with the colon carcinoma age-specific incidence data, while the multi-stage hypothesis is not. To determine this, I compared the mathematical representation of the parallel route hypothesis, Equation 3, with the age-specific incidence data. In the same manner, I also compared the mathematical representation of the multi-stage hypothesis (the Armitage-Doll model Equation 2 [4]) with the age-specific incidence data. See Figure 1. (Figure S1 demonstrates that the computer simulations accurately represent the Armitage Doll model.) The specific results are that the probability one should accept the parallel route hypothesis is 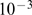 (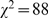, with 52 degrees of freedom), while the probability that one should accept the multi-stage hypothesis, Equation 2, is 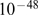 (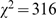, with 53 degrees of freedom), see Figure 1.

The measured values characterizing the fit of Equation 3 to the data, 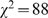 and 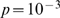, are somewhat high. This is partly caused by a slight, but significant, excess of colon carcinoma cases that occurred at ages 65 and 66. This excess is probably due to the increased access to medical care when Medicare eligibility begins at 65. If the excess cases at ages 65 and 66 are removed, the values characterizing the fit drop to 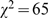 and 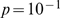. In the SEER-9 data, which is less powerful, this effect is not noticeable.

I also compared the colon carcinoma data to the exact representation of the multi-stage hypothesis, Equation 1. This formulation has three parameters just like the formulation of the parallel route hypothesis. I found that one should not accept this formulation either, (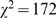, with 52 degrees of freedom, 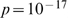).

The mathematical equation (Equation 3) of the parallel routes hypothesis has three parameters, and each has a well defined meaning. The first parameter, 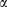, describes the fraction of the total population susceptible to this carcinoma. This parameter is always greater than the actual fraction who develop the disease. The parameter 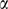 can vary from 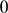 to 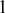. The second parameter, the mean time, 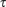, indicates the average time, measured from birth, for the formation and detection of the cancer. This is a theoretical average, the actual average will always be less, since many die from other causes before getting cancer. One would expect that environmental influences can affect this parameter, and that may be one explanation for the variation of times observed in the population. The third parameter, the standard deviation of the time, 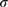, quantifies this variation in the population. This variation can be attributed to either intrinsically random processes, genetic variation, or environmental differences within the population. It is also a function of the number of parallel routes, 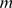, as shown in Scheme 2 and Figure S2.

Surprisingly, the age-specific incidence data implies that only about one of every five could ever develop colon carcinoma through this process. This suggests the existence of two distinct sub-populations that are determined either before birth or at a very young age. One subpopulation is destined to develop colon carcinoma, while the other will never develop it. Competing risk, or death due to other diseases, does not confuse this interpretation, since this age-specific data corrects for reductions in the population.

For the next test of the hypothesis, I used the SEER-9 data [17] to perform 31 similar tests (one for each year from 1973 to 2003) on the four most common types of carcinoma: lung, colon, breast, and prostate carcinoma. The SEER-9 data monitors fewer people than the SEER-17 data, but has been collected since 1973. Using this data allows more independent tests of the hypothesis.

**Figure 2:**
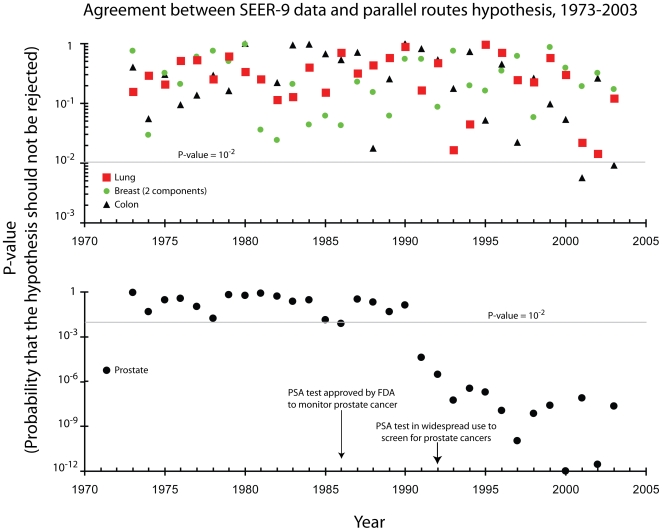
This graph quantitatively shows the agreement between the SEER-9 data and the hypotheses (Equations 3 and 4). The top panel displays the agreement, as measured by a p-value, between lung and colon carcinoma and Equation 3, and between breast carcinoma and Equation 4. In all cases the p-value, representing the probability that one should accept the hypothesis, is greater than 0.001, and in most cases it exceeds 0.01. In contrast, the corresponding graph for prostate carcinoma and Equation 3 shows that the p-value always exceeded 0.01, until 1991 when it plunged below that level. Prostate carcinoma, post 1991, clearly cannot be explained by Equation 3, but it is in agreement with Equation 4. The 1991 change corresponds to the widespread implementation of screening for prostate carcinoma using the PSA test.

**Figure 3:**
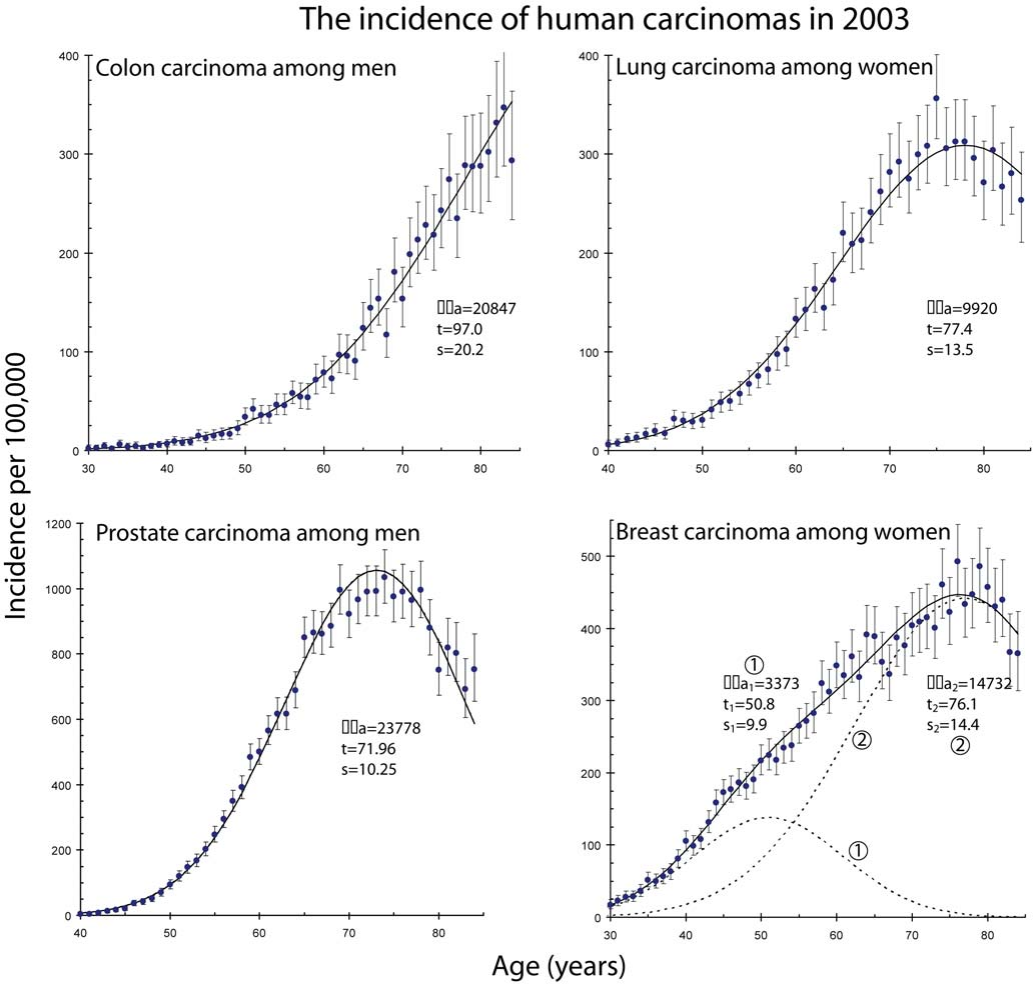
This is a comparison between the hypotheses and the observed age-specific incidence of colon, lung, prostate, and breast carcinomas in 2003 as recorded by the SEER-9 registries. In each case, the measured incidence is represented by a point and the 95% confidence intervals by error bars. A solid line represents the hypothesis, (Equation 3 for colon, lung, and prostate carcinomas and Equation 4 breast carcinomas) and the parameters for the model are indicated on the graph.

The parallel routes hypothesis was consistent with the age-specific incidence date for lung and colon carcinomas for all 32 years. However, the results for prostate and breast carcinoma were more complicated. See Figure 2. Detailed graphs of the data, along with the parallel routes hypothesis, are shown for 2003 in Figure 3. A table presenting the fraction of the population that is susceptible to the disease is shown in Table 1.

**Table 1:** This table shows an estimate of the fraction of the population susceptible to each form of carcinoma, as measured by the parameter 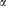.

Tissue	Susceptible population ( 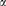 )	Lifetime probability
Colon	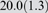 %	5%
Lung (female)	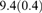 %	6%
Lung (male)	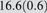 %	8%
Breast A	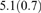 %	–
Breast B	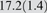 %	12%
Prostate	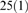	16%

The estimate is given as the mean value measured in five recent years, with the standard deviation given in parenthesis. It also gives the lifetime probability of developing carcinoma for all races in the 17 SEER areas over the period of 2004–2006. (The total lifetime probability of developing breast carcinoma is 12%.)

Prostate carcinoma age-specific incidence data was consistent with the parallel route hypothesis until 1991, see Figure 2. The 1991 time period corresponds to the widespread adoption of the PSA serum test for prostate cancer screening. The PSA test was first approved in 1986, but it was initially used only to monitor the progress of prostate cancer patients. In 1991 a study [18] showed that the best method of screening for prostate cancers is measurement of serum PSA levels combined with digital rectal exams. This radically changed the diagnosis procedure for prostate carcinoma.

The age-specific incidence data measures the time from birth to the diagnosis of carcinoma. This time includes two distinct components. The first is the time from birth to the development of the carcinoma. The second is the time from development of the carcinoma to its diagnosis. For prostate carcinoma, the second component is long; many prostate cancers grow slowly. The introduction of widespread screening has substantially shortened the second component of the time and thus changed the age-specific incidence data.

Breast carcinoma has never (1973–2003) been consistent with Equation 3. Instead, breast carcinoma could only be consistent with the parallel routes hypothesis, if one assumes that **three** different sub-populations exist, as expressed in Equation 4. One of these sub-populations is not susceptible to the disease, while the other two sub-populations can develop breast carcinoma. These distinct sub-populations probably develop distinct forms of the disease.

Other studies also suggest that two distinct forms of breast carcinomas exist. Early-onset breast carcinoma is already a recognized subclass of the disease, typically being described as occurring in women before the age of 35 [19]–[21]. My results indicate significant overlap between the early and late onset forms, and age itself is insufficient to determine whether a woman has one form of the disease or another. (Hence, I refer to these as breast carcinoma **A** and breast carcinoma **B**). However, most cases (about 80%) diagnosed before the age of 35 will be breast carcinoma **A**. Early-onset disease is biologically distinct [22], [23] from the late onset version and also a more potent form of the disease [24], [25]. Furthermore, recent work comparing the age distribution patterns for different histopathologic types of breast cancer using smoothed density plots [26], [27] found results similar to this [28], including a bimodal distribution of age at diagnosis, with one mode centered about 50 years, and the second about 70 years. Hence, my conclusion is consistent with an emerging view of breast cancer [21].

The origin of these two distinct sub-populations that develop breast carcinoma is unclear. One possibility, these correspond to inherited and sporadic cancers, seems unlikely. Three of 211 (95% confidence intervals, 0%–7.2%) breast cancer patients in a population based study were found to have inherited mutations in BRCA1 [29]. These small numbers of BRCA1 mutation breast cancers are unlikely to be apparent in this data.

Finally, I applied this hypothesis to better understand the racial disparity in breast cancer. This striking disparity exists in the prognosis for breast cancer patients in the United States. Although African-Americans are less likely to contract the disease, a significantly larger percentage of these patients die from it, compared to white patients. Furthermore this gap has been increasing over the past few decades [30]. While the obvious cause, unequal treatment, may be responsible for some of this disparity, it is not responsible for all. A detailed study of over 20,000 breast cancer patients treated in the equal access Department of Defense Health care system between 1980 and 1999 also revealed a consistent and growing disparity [31].

I reasoned that since early-onset breast cancer is known to be more deadly [24], [25] and that 80% of early-onset cases are breast carcinoma **A** then perhaps the racial disparity exists because African-American women may be less likely to develop breast carcinoma **B**. To test this, I extracted age-specific incidence data for the year 2000 for both African-American and white women from the SEER-17 dataset. I simultaneously fit the mathematical equation of breast carcinoma age-specific incidence (Equation 4) to each data set. I used the constraint that the breast carcinoma **A** parameters were identical for both African American and white women, while the breast carcinoma **B** parameters could vary.

**Figure 4:**
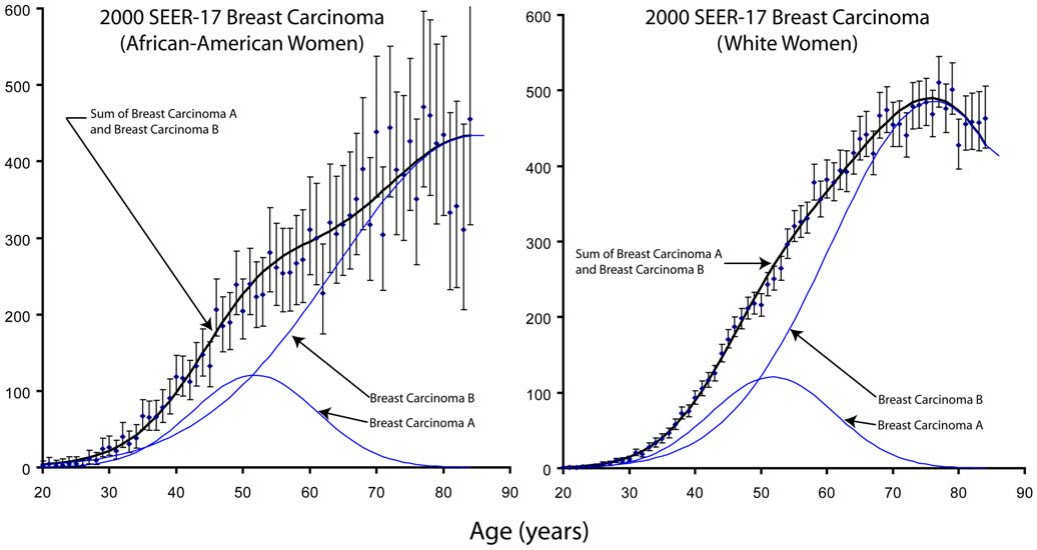
The breast carcinoma disparity between African-American and white women is not due to breast carcinoma A, which occurs at exactly the same rate, but solely due to a difference in breast carcinoma B. The data are from the SEER-17 database during the year 2000 [17]. In each case, the measured incidence is represented by a point and the 95% confidence intervals by error bars. The dark solid lines represents predicted incidence levels based upon the hypothesis, Equation 4, which is the sum of breast carcinoma A and breast carcinoma B indicated by the other curves.

I found that the age-specific incidence is consistent with the hypothesis that breast carcinoma **A** has no racial disparity, while breast carcinoma **B** incidence has substantial racial disparity. See Figure 4. Breast carcinoma **B** accounts for three quarters of the cases of breast cancer in the United States. A test that could distinguish breast carcinoma **B** from breast carcinoma **A** might give promising prognosis information to many patients.

The results presented here indicate that the age-specific incidence data for most human carcinomas is consistent with the parallel route hypothesis as expressed in Equations 3 and 4. The parallel routes hypothesis, then, implies (through the age specific-incidence data) that only a subset of the population is susceptible to developing each carcinoma. Other hypotheses may be consistent with this equation, but the implications are not dependent upon the hypothesis.

These implications, or predictions, can be tested. First, the age-specific incidence of breast and prostate carcinomas should decrease for those over 85 years of age. Second, cancer incidence in international populations should follow the same equation here. Finally, unlike prostate and colon carcinomas, breast carcinomas form in two fundamentally different ways.

This hypothesis may provide useful guidance for whole genome analysis of carcinomas. The genetic basis for some diseases [32] have been identified using whole genome analysis, but it has not shown similar success when applied to carcinomas [33], [34]. Whole genome analysis compares genomes of those who have cancer with those who do not. At least two complications arise in this analysis. First is the proper identification of samples without cancer, since it is not possible to identify people who will never be diagnosed with cancer. Second is that samples are usually drawn from peripheral blood; these would only be sensitive to germ-line mutations.

Two changes in the approach to whole genome analysis might be needed. First, an independent measure of the population that will not develop carcinomas is needed. This analysis can provide such a measure. Second, DNA samples need to be drawn directly from the relevant tissue. Only about 40% of children who develop retinoblastoma have a germline mutation [35]. If these statistics are true for carcinomas, the current approach to whole genome analysis will not be successful.

Finally, similar conclusions have been drawn by others [36]–[39] using different methods. Other work on age-specific incidence has focused on the two stage with clonal expansion model [40]–[44] and on developing novel techniques for the analysis of this data [8], [45], [46]. Different mechanisms could explain the existence of a sub-populations susceptible to carcinoma. For instance, members of this sub-population may have inherited susceptibility conferred through low-penetrance alleles [47] or may have acquired a somatic mutation early in life [48].

In conclusion, the parallel routes hypothesis is consistent with the age-specific incidence data for most common forms of human carcinoma. Furthermore, the age-specific incidence data suggest that only a measurable sub-population is likely to contract carcinoma.

## Materials and Methods

### Mathematical Models

The multi-stage hypothesis implies that the cancer age-specific incidence should follow a Poisson process. The differential form of the Poisson process is,
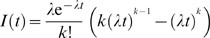
(1)where 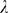 is the expected number of events that occur per unit time and 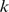 is the number of rate limiting events required before cancer occurs. However, the age-specific incidence is typically modeled by the Armitage Doll equation [4],
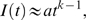
(2)where 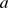 is an arbitrary constant and 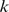 is again the number of rate limiting events required before cancer occurs. This is an approximation, valid for 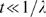, of Equation 1. The condition 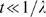 is equivalent to saying that only a small percentage of the population develops cancer.

The parallel route hypothesis is a combination of an unknown number of Poisson processes. Thus, the Central Limit Theorem suggests that the age-specific incidence, 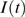, of carcinoma for the parallel route hypothesis is given by
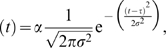
(3)where 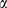 represents the fraction of people susceptible to the cancer. This is the well-known normal distribution, also called the Gaussian distribution, or bell-shaped curve. The Supplemental Information S1 shows that the application of the Central Limit Theorem is appropriate when the number of parallel routes is about 10. (An exact solution requires that 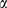 be reduced by the fraction of people who have already developed cancer, see [49]. Since carcinoma occurs in a small percentage of the population, as opposed to cancers in atomic bomb survivors, treating 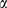 as a constant is an approximation good to better than 1%.)

If two distinct sub-populations can develop carcinoma through a different set of processes, the observed age-specific incidence will be a linear combination of two independent functions,
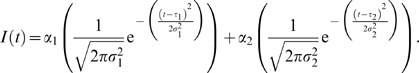
(4)


### Hypothesis Testing

To test the hypothesis that carcinoma age-specific incidence data follow Equation 2, or Equation 3, or Equation 4, two steps are required. First, the unknown parameters, (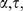, and 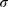, in the case of Equation 3) must be determined; I used maximum likelihood estimation to do so. Second, the probability that the observed data was generated by the postulated equation is determined. I used the chi-squared test for goodness-of-fit to determine this probability.

The maximum likelihood estimator was
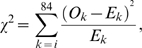
(5)where 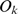 is the observed number of carcinoma cases and 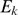 is the expected number of carcinoma cases in each of the 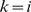 to 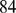 age ranges. The SEER dataset provides counts of cancer cases (and population data) in one year intervals from 0 to 84 years. It also includes data on all cases for those greater than 85 years old, this was excluded. The minimum age used, 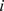 years, was chosen so that at least 10 cases were present in that year. It was typically in the 20s or 30s, depending on the cancer. The expected number of cases, 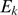, was obtained by multiplying the age-corrected incidence function 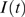, by the population under surveillance in that age range.

Once the parameters were estimated, the chi-squared value was determined, from Equation 5. A p-value, or probability that the fit should be accepted, was calculated based as the one-tailed probability of the chi-squared distribution.

I took several measures to ensure over fitting was not a problem. Over fitting can be caused by fitting an arbitrary mathematical function with multiple free parameters to a dataset. The defining characteristic of over fitting is the inability of the model to fit multiple independent datasets. The first step I took is to fit independent data sets from successive years. This gives me confidence that the model is representative of the underlying data.

### SEER Data

I tested the models, Equation 3 and Equation 4, by fitting them to age-specific incidence data from different carcinomas. The age-specific incidence data were recorded by the SEER 9 registries [17]. The SEER registries have compiled cancer incidence information on a large representative sub-population of US residents since 1973. From this database, I selected patients diagnosed in a particular year with carcinoma in the indicated tissue. This excludes the small number with other types of cancers, sarcomas for instance, which probably arise through a different process. The calculation of confidence intervals are based upon the method of Fay and Feuer [50].

I chose to analyze data for patients who were diagnosed in the same year (2000, for instance), rather than those born in the same year (a birth-cohort). Different factors could distort either data set. Birth-cohort analysis is significantly distorted by changes in medical practice and diagnostic technology. On the other hand, changes in environmental carcinogens may distort period analysis [42]–[44], like the data presented here. Detection technology for the carcinomas presented here have dramatically improved over the past 50 years. Hence, I focus on patients diagnosed in a single year, while recognizing that their environmental exposure may be different.

### Model Comparison

I used SEER-17 colon carcinoma data from 2000 for both men and women. This gave cancer cases and population under surveillance by individual years. I excluded those ages where fewer than ten cases were observed, ages less than 30. The data then included 22,344 cases in patients from 30 to 84 years of age, 55 independent data points. I tested the hypothesis that each was consistent with the data by first determining the parameters using the maximum likelihood method, then determining the goodness-of-fit by minimizing the 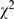 value.

## Supporting Information

Supplemental Information S1 A file containing the supplemental information. One page of results and methods describing Figures S1 and S2.
(0.04 MB PDF)

Figure S1 This graph presents the results of computer simulations based upon the assumption that carcinoma occurs through a single route, as depicted in Scheme 1. The points represent the simulation results, while the solid line represents Equation 1. The inset shows the log-log graph in the boxed region as the cancer age-incidence is usually shown.
(0.45 MB TIF)

Figure S2 This presents the results of computer simulations based upon the assumption that carcinoma may occur through multiple parallel routes. The four situations represent different assumptions in the computer simulation of 1, 5, 20, or 50 different routes along which carcinoma may occur. The solid lines represent the best fit to each, from Equation 3. The single route simulation, similar to that shown in Figure S1, is clearly not fit by the equation. The 5 parallel routes exhibit slight systematic deviations from the equation, but the 20 and 50 route assumptions are well described by the equation.
(1.26 MB TIF)
