# Supplementary material for: Parallel Routes of Human Carcinoma Development: Implications of the Age-Specific Incidence Data
Source: PLoS One. 2009 Sep 23;4(9):e7053. doi: 10.1371/journal.pone.0007053 (PMC2743810; doi:10.1371/journal.pone.0007053)
Supplement: Supplemental Information S1 — A file containing the supplemental information. One page of results and methods describing Figures S1 and S2. (0.04 MB PDF) [file pone.0007053.s001.pdf]

Supplement to **Parallel routes of human carcinoma development: Implications of the age-specific incidence data**

James P. Brody[1]
1 Department of Biomedical Engineering University of California–Irvine Irvine, California, USA 92697-2715
∗ **E-mail: jpbrody@uci.edu**

Results and Discussion

I performed computer simulations of Scheme 1 and Scheme 2 to confirm that Equation 1 is the appropriate mathematical representation of the multi-hit hypothesis (Scheme 1) and that Equation 3 is the appropriate representation of the parallel route hypothesis (Scheme 2).

The results of these computer simulations are presented in Figure S1 for the multi-hit hypothesis, and in Figure S2 for the parallel routes hypothesis. These results confirm that the appropriate mathematical representation was used. In the case of Scheme 2, the results also show the relative effect of the number of routes on the width of the age-specific incidence curve. Thus, Equation 3 is an appropriate representation of the parallel routes hypothesis.

Materials and Methods

Computer Simulations I wrote a computer program to simulate Scheme 1 and Scheme 2. The program tracked 100,000 subjects (people). For each subject, it kept a list of genes and routes. Each route contained 50 genes, for Scheme 2. For each time step, a random integer was chosen from 1 to 100 and a gene was said to be mutated if this number was equal to 1. If 50 genes were found to be mutated in sequence, the subject was recorded as having developed cancer at that time step. I chose 50 since recent sequencing studies have shown that 50-100 genes are mutated in some forms of cancer [1, 2]. Similar results can be obtained using different numbers of genes per route, mutation rates, and mutations required, although the time scales will be altered. I also investigated cases in which different routes are not equally probable and where mutation rates vary for different genes. In both cases, I obtained results similar to those in Figure S2. One complication that needs further study is that some genes are almost certainly present in multiple routes.

References

1. Jones S, Zhang X, Parsons DW, Lin JCH, Leary RJ, et al. (2008) Core signaling pathways in human pancreatic cancers revealed by global genomic analyses. Science .

2. Wood LD, Parsons DW, Jones S, Lin J, Sj?blom T, et al. (2007) The genomic landscapes of human breast and colorectal cancers. Science 318: 1108–1113.
