# Supplementary figures and images for: Parallel Routes of Human Carcinoma Development: Implications of the Age-Specific Incidence Data
Source: PLoS One. 2009 Sep 23;4(9):e7053. doi: 10.1371/journal.pone.0007053 (PMC2743810; doi:10.1371/journal.pone.0007053)

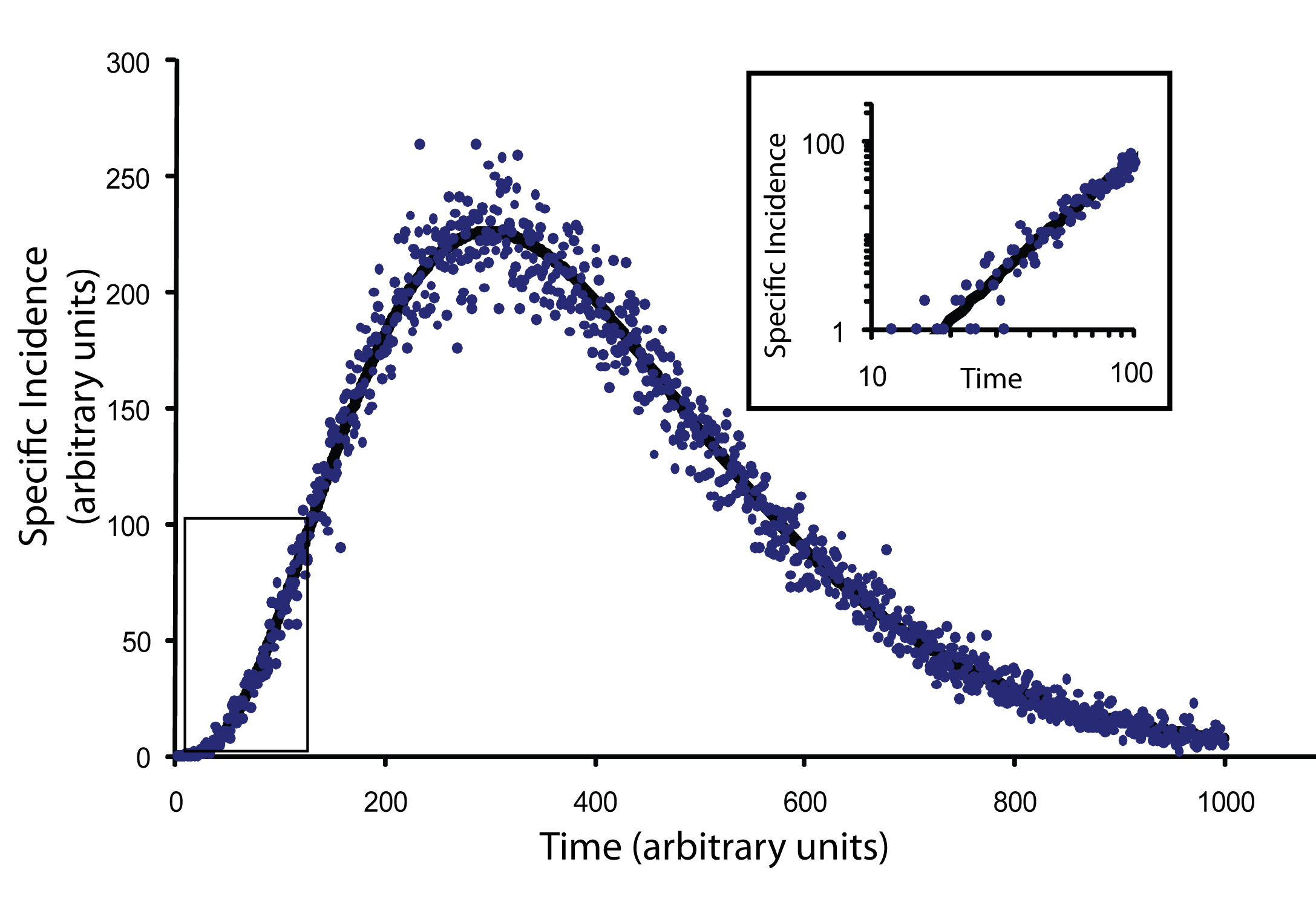

Supplement: Figure S1 — This graph presents the results of computer simulations based upon the assumption that carcinoma occurs through a single route, as depicted in Scheme 1. The points represent the simulation results, while the solid line represents Equation 1. The inset shows the log-log graph in the boxed region as the cancer age-incidence is usually shown. (0.45 MB TIF) [file pone.0007053.s002.tif]

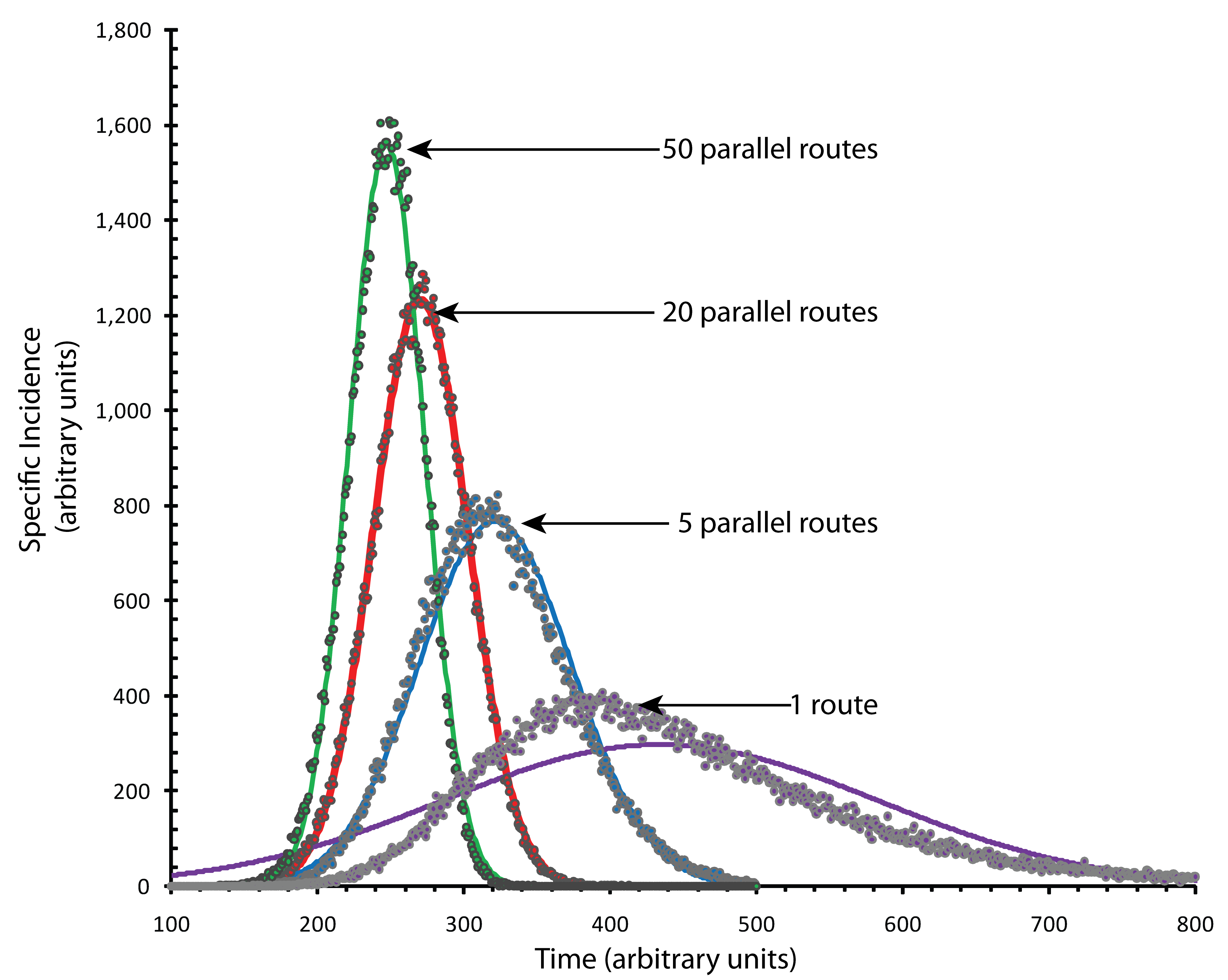

Supplement: Figure S2 — This presents the results of computer simulations based upon the assumption that carcinoma may occur through multiple parallel routes. The four situations represent different assumptions in the computer simulation of 1, 5, 20, or 50 different routes along which carcinoma may occur. The solid lines represent the best fit to each, from Equation 3. The single route simulation, similar to that shown in Figure S1, is clearly not fit by the equation. The 5 parallel routes exhibit slight systematic deviations from the equation, but the 20 and 50 route assumptions are well described by the equation. (1.26 MB TIF) [file pone.0007053.s003.tif]
